# Interoperable smart city transformation: Insights from European data space for smart communities

**DOI:** 10.1016/j.dib.2026.112677

**Published:** 2026-03-26

**Authors:** Sophie Meszaros, Anniki Puura, Thimo Thoeye

**Affiliations:** a Open and Agile Smart Cities (OASC), Rue du Luxembourg 19-21, Brussels 1000, Belgium; b imec-SMIT Vrije Universiteit Brussels Bd de la Plaine 9, Etterbeek 1050, Belgium; c FinEst Centre for Smart Cities, Tallinn University of Technology, Ehitajate tee 5, Tallinn 12616, Estonia

**Keywords:** Interoperability, Data space, MIMs plus, Smart cities, Smart communities

## Abstract

The European Data Space for Smart Communities (DS4SSCC) is a flagship initiative demonstrating how interoperable, cross-domain data infrastructures can drive smart city transformation in Europe. Yet achieving interoperability across legal, organisational, semantic, and technical layers remains difficult for local governments constrained by legacy systems, rigid procurement, and fragmented ICT landscapes. This paper examines how interoperability is operationalised within DS4SSCC through the Minimal Interoperability Mechanisms (MIMs) Plus framework. Using a qualitative case study approach— combining document analysis, stakeholder survey data, and workshop insights from the DS4SSCC Preparatory Action—the study analyses how governance structures, technical standards, and procurement mechanisms interact to enable cross-domain collaboration. The findings identify five key enablers of interoperability-by-design: modular data architectures, shared governance, lightweight semantic alignment, interoperability clauses in procurement, and capacity building. Foundational MIMs (Accessing, Representing, Interlinking, Securing, and Sharing Data) underpin DS4SSCC’s core building blocks, while application-specific MIMs (Personal Data, Geospatial, Interoperable AI, and Local Digital Twin) support more complex, cross-domain use cases. Persistent challenges include uneven capacity, vendor lock-in, and variable conformance maturity. The paper concludes that MIMs Plus translates the four layers of the European Interoperability Framework into actionable mechanisms, positioning interoperability as a continuous governance capability essential for scalable and trustworthy data spaces.

## 1 Background

### 1.1 Interoperability in the Digital Transformation of the Public Sector

Across Europe, the ongoing push for digital transformation has revealed persistent structural and institutional gaps in the public sector’s information and communications technology (ICT) ecosystems. Cities and communities increasingly rely on data-driven processes to enhance public services, foster urban innovation, and accelerate both the green and digital transitions. However, the transformative potential of these initiatives is frequently constrained by deeply fragmented digital infrastructures that limit the seamless exchange and reuse of data across organisational and jurisdictional boundaries [[1], [2], [3]]. Despite widespread acknowledgement of the value of interoperable systems for efficiency and innovation, many public administrations remain hampered by legacy architectures, inconsistent standards, and institutional inertia that impede the development of integrated digital ecosystems [4]. Integration across departments, sectors, and governance levels thus remains technically complex, financially burdensome, and legally constrained, often due to rigid procurement rules, incompatible data standards, and path-dependent administrative routines [[5], [6], [7]]. These structural and procedural constraints collectively undermine the capacity of local and national administrations to realise systemic interoperability—a prerequisite for scaling data-driven public governance and sustainable urban innovation [8,9].

Interoperability has therefore emerged as a foundational condition for realising the goals of data-driven governance and smart city transformation. Wegner’s (1996) [10] definition of interoperability as the “ability of two or more software components to cooperate despite differences in language, interface, and execution platform” captured the technical essence of the concept. However, subsequent research has substantially broadened this view, framing interoperability as an inherently socio-technical phenomenon shaped not only by technological architectures but also by institutional arrangements, governance mechanisms, and human capacities [[11], [12], [13]]. Earlier contributions, such as Misuraca et al. [14], already emphasised the need for a pan-European conceptual framework that combines semantic, technical, and organisational layers to address interoperability challenges in ICT-enabled governance, anticipating many of the multi-dimensional approaches later institutionalised through the European Interoperability Framework (EIF) in 2017 [[15], [16], [17]]. Achieving interoperability thus requires aligning legal rules, organisational processes, semantic vocabularies, and technical infrastructures – not just connecting systems – to foster meaningful cross-context collaboration and data reuse [18]. In urban innovation settings, such alignment becomes especially challenging because data is generated and managed by a wide array of actors – municipalities, private firms, utilities, and citizens – each governed by different institutional logics and regulatory constraints [19,17].

The European Union has increasingly recognised these multi-level dynamics, making interoperability a central pillar of its digital and urban transformation agenda. The 2017 EIF formalised the coordination of legal, organisational, semantic, and technical interoperability into a coherent governance approach that embeds policy considerations alongside technical design [15]. The establishment of the National Interoperability Framework Observatory (NIFO) further institutionalised this approach, tracking member states’ progress and fostering exchange of best practices across European administrations [20]. Parallel initiatives, such as the United Nations Integrated Geospatial Information Framework (UN IGIF), extend this logic globally, identifying governance, policy, finance, data, innovation, standards, partnerships, capacity-building, and communication pathways as pillars for integrated service delivery [21]. Complementary efforts within the EU have also highlighted the role of application programming interfaces (APIs) as foundational enablers of interoperability. According to Vaccari et al. [22], APIs serve as technical and policy instruments that allow governments to open data and services in structured, machine-readable forms, promoting transparency, innovation, and cross-border integration across European administrations. Together, these frameworks represent a paradigm shift from viewing interoperability as a purely technological issue to recognising it as a systemic governance challenge that demands sustained coordination, shared standards, and strategic investment [23,24].

Public procurement represents one of the most powerful, yet underutilised, policy instruments to advance interoperability objectives. With European public procurement budgets amounting to approximately €2 trillion annually – around 14% of the EU’s GDP – public authorities wield considerable leverage to embed interoperability requirements into digital system acquisitions [25]. However, procurement frameworks designed for risk aversion and accountability often constrain innovation and flexibility. As studies in digital government indicate, rigid procedures, limited cross-sector collaboration, and fragmented funding streams frequently prevent administrations from experimenting with modular, open-source, or standards-based solutions [5,8]. Buyle et al. [26] reinforce this argument by proposing sustainable methodologies for publishing interoperable open data on the web, demonstrating how open standards and linked-data technologies can reduce technical barriers while enhancing data reusability across public- sector platforms [27]. These challenges are compounded by a shortage of digital skills and strategic data governance capacities within local administrations, which hinders their ability to design, evaluate, and procure interoperable digital solutions [28,29].

The benefits of overcoming these barriers are nonetheless well-documented. Interoperable systems enhance efficiency in public spending, facilitate evidence-based policymaking, and support cross-sectoral innovation [25,9]. Furthermore, research on smart city data ecosystems shows that semantic interoperability—ensuring data meaning is preserved and shared across systems—does not necessarily depend on exhaustive data exchanges. Instead, well-structured, standardised datasets often suffice to enable meaningful integration [13,18,30]. The General Transit Feed Specification (GTFS) exemplifies this principle: by defining a simple yet standardised format for describing transit routes and times, it enables consistent data sharing among transport operators, app developers, and public authorities worldwide [11,31]. In this sense, interoperability can be seen as both a technical condition and a governance capability, requiring a balance between standardisation and contextual flexibility.

### 1.2 From Interoperability to Data Spaces

Recent research highlights that data interoperability in the context of smart cities increasingly depends on the creation of governed, federated data spaces—decentralised infrastructures that enable trusted, cross-organisational data sharing under common governance principles [[32], [33], [34], [35]]. Studies on data spaces [36,37] argue that these environments operationalise interoperability through data harmonisation, semantic mediation, and standardised governance models. For example, Diaz-de-Arcaya et al. [37] identify data harmonisation as the “keystone” of functional data spaces, emphasising the importance of aligning metadata, data models, and exchange protocols to ensure semantic and technical coherence. Complementary research by Sapienza et al. [2] and Gilman et al. [1] links these developments to the European Data Strategy, which aims to establish a single market for data through sectoral and cross- sectoral data spaces that balance openness, sovereignty, and trust. Within urban contexts, these principles are increasingly applied through digital twin and smart city initiatives, which rely on interoperable data infrastructures to simulate, monitor, and manage urban processes in real time [19].

In parallel, Martella et al. [38] provide an in-depth overview of the technical specifications guiding European data-space initiatives, identifying key convergence points among frameworks such as Gaia-X, IDSA, FIWARE, and iSHARE. Their analysis underscores that technical interoperability now relies on shared architectural blueprints, standard APIs, and governance agreements that ensure interoperability-by- design across heterogeneous ecosystems [39]. Similarly, the Data Spaces Business Alliance [40] provides detailed guidance on the technical and governance convergence shaping the European data space landscape, stressing the importance of common reference architectures and open-source enablers for cross-sector collaboration. This convergence between policy and technology demonstrates how interoperability is being reinterpreted as a mechanism for value creation and resilience in complex urban systems.

The emerging literature also reveals growing attention to the socio-organisational dimensions of interoperability. Fassnacht et al. [8] show that successful data-sharing practices depend on the interplay between data quality, organisational structures, and network dynamics rather than on technology alone. Similarly, Bozkurt et al. [4] benchmark various data governance models for smart cities, concluding that hybrid governance approaches – combining central coordination with decentralised execution – best accommodate Europe’s diverse urban contexts. These insights align with broader discussions on semantic- artefact governance [41] and the need for adaptable frameworks that can evolve alongside technological and regulatory changes. As Hoang [42] notes, ensuring high-quality, harmonised data remains a persistent challenge that directly affects the usability and trustworthiness of shared data systems.

In practice, achieving such quality requires continuous validation and coordination between data producers and consumers—a task often underestimated in urban innovation projects. This challenge is also reflected in recent policy documents and technical initiatives from the Digital Europe Programme [43], Data Space Support Centre (DSSC) [44], and Gaia-X [45], which highlight the critical role of certification mechanisms, trust frameworks, and interoperability testbeds for advancing cross-border data exchange within the EU’s digital ecosystem. Within this policy and research landscape, the EU’s evolving regulatory architecture – especially the Data Governance Act [46] and Data Act [47] – has consolidated interoperability as a legal and technical mandate for data-driven transformation.

The Data Act [47] in particular mandates that Common European Data Spaces [48] embed not only interface standards but also governance, trust, and enforceability mechanisms. Among these, the European Data Space for Smart Communities (DS4SSCC) [49], funded under the Digital Europe Programme, is especially significant as it bridges public administrations, industry, and research institutions to facilitate cross-domain interoperability in smart communities [2,39]. The DS4SSCC initiative offers a living laboratory to observe how interoperability frameworks are implemented across levels – from local to European – in practice, revealing how technical standards, governance models, and procurement processes interact within a multi-level, multi-stakeholder ecosystem [36].

Empirical studies indicate, however, that operationalising interoperability within such complex arrangements remains a formidable task. Path dependencies in public ICT procurement, fragmented governance structures, and limited administrative capacity continue to pose significant obstacles [5,8,7]. Moreover, political debates around digital sovereignty and control over data infrastructures add an additional layer of complexity, as member states seek to balance openness with strategic autonomy [50]. Yet, as Nicholson et al. [23] demonstrate in the context of health-data spaces, trusted data-sharing architectures can emerge when institutional mechanisms and technical protocols are co-designed with transparency, accountability, and reusability in mind. Translating this logic to smart communities implies that interoperability is not a static feature but a dynamic capacity for continuous coordination among diverse actors [8,1].

Against this backdrop, the DS4SSCC provides a crucial empirical case to study how interoperability is enacted across institutional boundaries and how governance and technical principles co-evolve in practice. Despite extensive conceptual and policy work, significant gaps remain in understanding how interoperability is implemented, governed, and sustained over time in the public sector. Addressing these gaps is essential for both theory and practice: theoretically, it advances the conceptualisation of interoperability as a socio-technical and governance phenomenon; practically, it informs policymakers and practitioners on enabling conditions for sustainable and scalable data exchange in smart urban ecosystems [28,37]. Accordingly, this research asks:

1. How is interoperability operationalised within the European Data Space for Smart Communities (DS4SSCC)?
2. Which principles support the implementation of interoperability in public-sector data spaces?

To address these questions, the study employs a qualitative case-study design, combining document analysis, stakeholder surveys, and workshop outputs. It analyses how governance structures, procurement practices, semantic models, and technical standards interact in practice and maps these empirical observations onto the four layers of the European Interoperability Framework—legal, organisational, semantic, and technical—to infer principles that support sustainable and scalable interoperability in the public sector. This approach contributes to the literature on smart city governance, digital transformation, and interoperability by bridging conceptual frameworks with real-world implementation dynamics. The following section outlines the methodological design adopted to explore these relationships and to derive actionable insights for both researchers and practitioners engaged in Europe’s evolving data-space ecosystem.

## 2 Methodology

### 2.1 Analytical Lens: MIMs Plus as a Framework for Interoperability

To analyse how interoperability is operationalised in DS4SSCC, this study builds on the Minimal Interoperability Mechanisms (MIMs) Plus framework as an analytical lens. The MIMs Plus framework promotes agile and modular interoperability, enabling public administrations to improve data exchange, integrate legacy systems, and design new interoperable solutions from the ground up via a catalog of compliant mechanisms and technical specifications. Developed under the Living-in.EU initiative [24] and coordinated by Open and Agile Smart Cities (OASC) [51]. The structure of the MIMs Plus framework, now at Version 8.0 [52], has been shaped through a collaborative, needs-driven process involving the MIM Working Groups and the Living-in.EU Technical Working Group. This co-evolution is documented in the DS4SSCC Strategy Report, which includes how survey and workshop inputs feed into refining MIMs definitions and priorities [53].

**Fig. 1:**
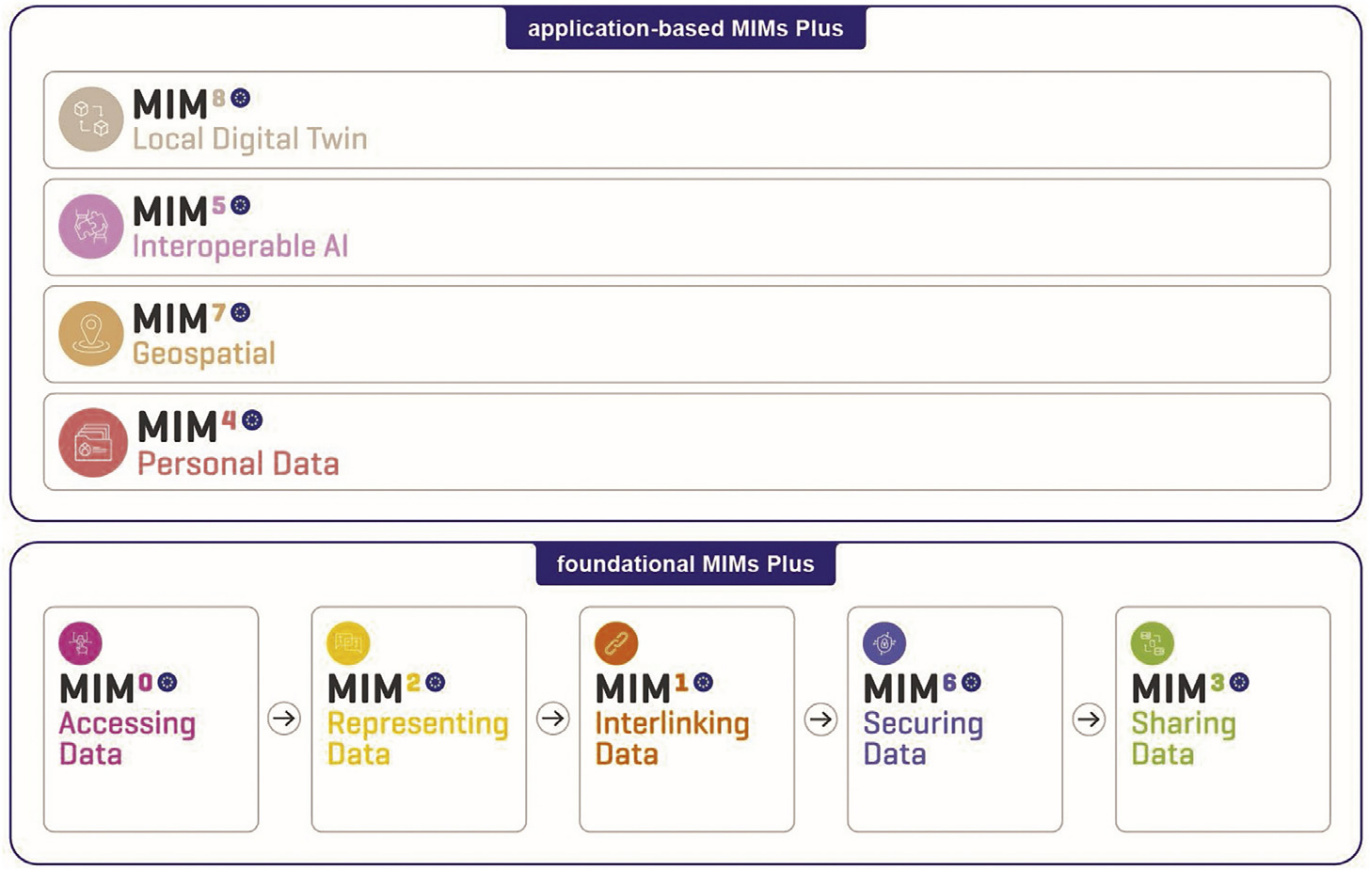
MIMs plus framework overview as published in the minimal interoperability mechanisms (MIMs Plus) - (Intermediate) Version 8 [52].

As shown in Fig. 1, the framework distinguishes between foundational mechanisms (Accessing, Representing, Interlinking, Securing, and Sharing Data) that address the data journey, and application- specific mechanisms (Personal Data, Geospatial, Interoperable AI, and Local Digital Twins) that address domain-oriented interoperability challenges requiring additional governance and technical coordination [52]. To support consistent use of the individual MIMs, the ITU-T Y.4505 standard was developed and adopted at the Global Standards Summit (2024) [54]. According to Y.4505, a MIM should clearly define its objective, specify the required capabilities, list compliant technical specifications, and outline guidance for interoperability, conformance, and compliance testing.

In this paper, MIMs Plus is used methodologically to map interoperability practices within DS4SSCC against the four EIF layers. This analytical use enables systematic evaluation of how DS4SSCC’s architecture, governance, and data management mechanisms collectively advance interoperability.

**Foundational MIMs**

• MIM Data Access (Formerly known as ‘MIM 1 – Context Information Management’): Simplifies access to cloud-based or on-premise data sources.
• MIM Interlinking Data (Formerly known as ‘MIM 1 Context Information Management’): Enables seamless connection of datasets through shared ontologies and identifier management.
• MIM Representing Data (Formerly known as ‘MIM 2 Data Models’): Establishes consistent, machine-readable data definitions that allow reliable combination of datasets from multiple sources.
• MIM Securing Data (Formerly known as ‘MIM 6 Security’): Provides guidance for secure and trustworthy data exchange.
• MIM Sharing Data (Formerly known as ‘MIM 3 Contracts’): Defines contractual and technical mechanisms for exchanging data across actors, including the creation of local data marketplaces.

**Application-Level MIMs**

• MIM Personal Data (Formerly known as ‘MIM 4 Trust’): Focuses on user-centric data management and sovereignty.
• MIM Geospatial (Formerly known as ‘MIM 7 Places’): Facilitates consistent use and sharing of spatial and temporal data across domains.
• MIM Interoperable AI: Addresses interoperability challenges in deploying AI-based services across cities and regions.
• MIM Local Digital Twin: Supports integration of multidimensional data streams into visual and analytical models of the urban environment.

By accommodating a wide range of use cases and maintaining a strong focus on community needs, the MIMs Plus can be applied across three recurring scenarios: (i) technology procurement with capability consistency; (ii) cross-mechanism interaction and data exchange; and (iii) development and utilisation of interoperable solutions [55] (see more from Table 1).

**Table 1:** Scenarios, explanations and explanations of using MIMs in DS4SSCC context.

**(i) Technology procurement with capability consistency**	In this first scenario, local administrations use MIMs Plus to modernise legacy systems while preserving essential data and functionalities. Applying MIMs reduces integration work by standardising transformation and migration processes.	A local administration would like to update its Customer Relationship Management (CRM) System but would like to keep the same capabilities to avoid having to integrate all the information. In this process they would need to access and import historical data and transform said data for mapping it onto the new tool, Following the MIMs Plus recommendations, efforts pertaining to integration is minimised.
**(ii) Cross- mechanism interaction and data exchang**e	In the second scenario, interoperability across mechanisms rely on MIMs Plus guidance for data subscriptions and event management. Implementing MIM Accessing Data and MIM Interlinking Data improve the consistency and continuity of data exchange across departments.	A local administration would like to develop an air quality drop notification service and for this, they need to import data from the National Environmental Agency. For this, the local authority needs to subscribe to notifications, which requires context information management. An infrastructure following the MIMs Plus recommendation enables this.
**(iii) Development and utilisation of interoperable solutions**	In the third scenario, new services are designed according to MIMs Plus recommendations to ensure that data is machine-readable and reusable. This proactive application of MIM Representing Data and MIM Sharing Data supports scalability and replicability in other municipalities.	The local authority is developing a mobility service regarding parking availability. For this, they need to integrate data from different parking providers which should be machine readable. The MIMs Plus provide guidance for such instances.

### 2.2 Data Sources & Analysis Approach

This research adopts a qualitative case study design to explore how interoperability is achieved within the European Data Space for Smart Communities. The DS4SSCC serves as a representative and information- rich case of a public-sector data space initiative, encompassing both technical and governance components of interoperability. By using MIMs Plus as a coding and interpretive framework, the analysis links empirical observations to established European interoperability dimensions. We triangulate across three main data sources:

1 **Document analysis**: Publicly available DS4SSCC deliverables (reports, blueprints, and policy guidance documents) were reviewed, including the Multi-Stakeholder Governance Scheme, Architecture Model, Use Cases and Priority Datasets, Capacity Building Programme, and Strategy Report (see more about the content in Table 2). These documents encode intended governance, technical architecture, capacity strategies, and deployment logic.

**Table 2:** DS4SSCC deliverables used in document analysis.

Document Title	Content Description
Multi-StakeholderGovernance Scheme [57]	Delineates roles, principles, data cooperation mechanisms, legal frameworks, and strategic recommendations aimed at fostering trustworthy and interoperable data ecosystem development at both local and European levels.
Catalogue of Specifications [39]	A list of technical specifications to realise Data Space building blocks.
Architecture Model [58]	Presents a comprehensive architecture model outlining system components, interfaces, and their interactions to support the DS4SSCC project’s design vision.
Data Space Blueprint and Priority Data Sets [59]	A list of European use cases demonstrating cross-domain data sharing provide input for defining priority datasets and gaps, technical overlaps and specifications, common challenges and the use of minimal interoperability mechanisms.
Roadmap for implementing a European data space for smart and sustainable cities and communities [60]	A detailed roadmap and action plan for deploying a European data space dedicated to smart and sustainable cities and communities, covering governance, technical architecture, maturity phases, capacity building, and standardisation across local to European scales.
Capacity Building Programme [61]	The programme details the assessment of stakeholder needs, the design of a tailored training curriculum, and the implementation of mutual learning events to support the development of data spaces in communities.
Strategy report [53]	The strategy for the initiative, detailing its governance, technical blueprint, stakeholder engagement, roadmap, and deployment plans for a pan-European data space for smart and sustainable cities.
2 **Survey data**: DS4SSCC conducted a pre-forum online survey in February-March 2023 to assess capacity building needs among DS4SSCC Stakeholder Forum (preparatory action) members. The survey yielded 85 responses covering interest in training topics, preferred formats, and thematic priorities [56]. The responses were received from participants across 22 European countries, respondents including city officials, public administration staff, ICT vendors, standards organisations, researchers, and representatives of civil society and industry stakeholders.
3 **Stakeholder Forum outputs**: DS4SSCC Stakeholder Forum meeting outputs from March 2023 in the form of structured notes, mapping exercises, and plenary discussions provided further qualitative insights into the alignment of data space building blocks and interoperability mechanisms. In this meeting, participants collaboratively mapped core components against the Data Space Support Centre (DSSC) reference architecture.

### 2.3 Analysis Approach

A combined inductive–deductive coding strategy was applied: (1) **Inductive coding** allowed additional themes to emerge naturally from the material, such as issues around procurement, capacity building, or data model alignment that were not predefined in the EIF. This made it possible to link what the EIF suggests for achieving interoperability with what is actually addressed in the DS4SSCC context. (2) **Deductive coding** was guided by the four layers of the European Interoperability Framework (EIF) – legal, organisational, semantic, and technical – and by the MIMs Plus framework thematic areas (e.g.: accessing data), which provided a structured lens for reviewing the data. After coding, themes were grouped and compared to assess how they aligned with the EIF layers and MIMs Plus mechanisms.

The first stage aimed to understand how interoperability is supported in the DS4SSCC framework. The second stage focused on assessing the use and maturity of specific MIMs for realising the DS4SSCC building blocks. Evidence from documents, surveys, and workshops was systematically mapped against the individual MIMs Plus specifications to identify: how each foundational and application-level MIM is applied in practice; the operational outcomes (e.g., improved data accessibility, strengthened security, or cross-domain integration); and the relative maturity levels of the individual MIMs. Patterns of MIM related considerations were then compared across data sources to assess consistency and identify gaps, dependencies, and maturity differentials between foundational and application-level mechanisms. This mapping enabled a structured interpretation of how the MIMs Plus framework functions as both an analytical reference and a practical interoperability toolkit in DS4SSCC.

**Fig. 2:**
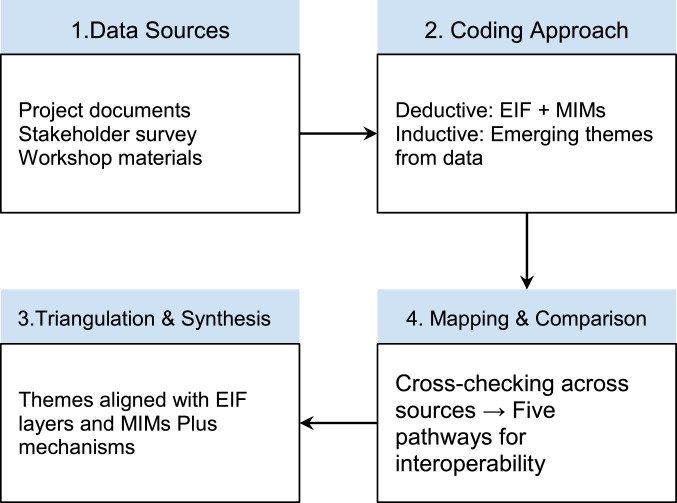
Framework for data analysis.

Findings from both stages were triangulated across the three data sources to ensure consistency and strengthen the credibility of interpretations. Manual coding and synthesis allowed close engagement with the material and transparent traceability from source data to analytical insight Fig. 2.

### 2.4 Ethical Considerations

The study adhered to established ethical guidelines for secondary data analysis and stakeholder engagement, ensuring compliance with the General Data Protection Regulation (GDPR) principles. All stakeholder materials used in this analysis – survey responses, workshop notes, and project documentation – were collected under informed consent procedures facilitated through the DS4SSCC project channels. Personal or identifying information was anonymised prior to analysis, and only aggregated insights were reported to maintain participant confidentiality. The research did not involve the collection of new personal data but relied entirely on materials produced within the project’s operational framework. Ethical oversight was provided by the DS4SSCC coordination team, under the supervision of the Digital Europe Programme (DEP) funding agreements. This ensured that data use, storage, and reporting were consistent with the ethical and legal obligations of EU-funded research.

## 3 Results & Analysis

The coding resulted in themes that were grouped and compared to assess how they aligned with the EIF layers and MIMs Plus mechanisms. This process revealed five recurring operational pathways through which interoperability was realised in DS4SSCC: modular data architecture, shared governance, lightweight semantic alignment, interoperability-oriented procurement, and capacity building. These pathways formed the analytical backbone of the first results chapter (Section 3.1).

### 3.1 Operational Pathways for Interoperability Within the DS4SSCC: EIF Principles

Drawing on triangulated evidence from document analysis, survey responses and workshop materials collected during the DS4SSCC Stakeholder Forum, the thematic analysis identified five recurring operational pathways through which interoperability was realised in practice. Mapped against the European Interoperability Framework’s legal, organisation, semantic and technical dimensions, the pathways operationalise interoperability.

1. **Modular data architecture**: Across DS4SSCC documentation and workshop discussions, participants consistently emphasised the importance of a modular, decentralised data architecture. This structure separates data publication, discovery, and exchange functions, allowing local administrations to integrate legacy systems incrementally while ensuring compliance with open interface specifications aligned with the Data Space Support Centre (DSSC) reference architecture. This modularity supports the EIF’s technical interoperability layer’s ambition for technological neutrality and adaptability and embodies the MIMs Plus emphasis on open and reusable components as a foundation for sustainable data space development.
2. **Shared and transparent governance**: The Multi-Stakeholder Governance Scheme established under DS4SSCC provides a clear division of roles among data providers, controlled, and consumers. Survey respondents and workshop participants reported that clarified governance roles and shared accountability mechanisms improve coordination and trust, especially in cross- border collaboration. The governance model embeds the FAIR principles (Findable, Accessible, Interoperable, Reusable) as a normative foundation, promoting transparency, inclusivity, and data stewardship. By institutionalising FAIR as part of governance design, DS4SSCC strengthens the EIF’s organisational interoperability layer (e.g.: openness, transparency, defined roles) and fostered trust in shared data infrastructures.
3. **Lightweight semantic alignment**: Rather than seeking full model harmonisation, DS4SSCC participants advocated for a “just enough” semantic approach – adopting minimal, reusable data models and shared identifiers. This approach facilitates efficient data integration across heterogeneous sources and preserves local flexibility while ensuring sufficient semantic interoperability. This resonates with the EIF Technical dimension’s motivation for interconnecting systems, integration and interface specifications, and scalability and flexibility.
4. **Procurement as a lever for interoperability**: Survey evidence and stakeholder discussions underscored procurement as a critical policy instrument for embedding interoperability. Incorporating interoperability clauses – such as requirements for open APIs, standard data formats, and data export functions – into tenders was reported to reduce vendor lock-in and lower integration costs. Document analysis confirmed that interoperability-by-design procurement practices are among the most effective institutional tools for implementing the EIF’s legal and organisational interoperability layers.
5. **Capacity building for sustainable implementation:** Stakeholders identified a strong demand for targeted capacity building on data space implementation, focusing on governance design, technical architecture, and data ethics. Both survey and workshop findings indicated a preference for case-based, practice-oriented learning formats, such as best-practice guides, webinars, and peer exchanges. Participants also highlighted the need for sustainable funding models to support long-term upskilling and for the inclusion of underrepresented groups in digital governance roles. These findings show that capacity building is not an auxiliary activity but a core enabler of interoperability maturity, directly enforcing the EIF’s organisational and legal layers towards building a shared cultured and common understanding.

Together, these five pathways demonstrate that DS4SSC treated interoperability not as a fixed technical output but as an evolving governance and capability framework. By combining architectural modularity, transparent governance, pragmatic semantics, procurement reform, and targeted capacity building, the initiative translated theoretical interoperability principles into operational mechanisms.

### 3.2 Principles Supporting Interoperability in the Public Sector: The Use of Specific MIMs in Data Spaces

Building on the thematic pathways identified in Section 3.1, this section presents the findings from the second stage of analysis, which examined how individual Minimal Interoperability Mechanisms (MIMs) were applied and matured within the DS4SSCC framework. Evidence from project documentation, survey responses, and workshop outputs was systematically mapped against each MIMs Plus specification to assess both operational implementation and institutional maturity. The analysis shows that while all MIMs contribute to interoperability development, their maturity and integration levels vary.

**Foundational MIMs**

• **MIM Data Access** (*Formerly known as ‘MIM 1 – Context Information Management*’): Supports the “data exchange” and “publication and discovery” building blocks. Documented evidence shows that adopting this MIM improves data accessibility and reduces onboarding time for new providers, particularly by standardising APIs and metadata exposure across local platforms.
• **MIM Interlinking Data** (*Formerly known as ‘MIM 1 Context Information Management’*): Survey and workshop feedback highlighted this MIM as critical for enabling cross-departmental data reuse without requiring full model harmonisation. It facilitates interoperability between legacy and new systems, strengthening operational efficiency and data consistency.
• **MIM Representing Data** (*Formerly known as ‘MIM 2 Data Models’)*: Workshop participants reported that the use of this MIM improves data accuracy and interpretability across city departments by promoting shared vocabularies and common data model templates, enhancing semantic interoperability.
• **MIM Securing Data** (*Formerly known as ‘MIM 6 Security’*): Stakeholders consistently emphasised this MIM’s role in reinforcing data sovereignty and GDPR compliance, which were identified as preconditions for trust in local and cross-border data sharing. This MIM operationalises the EIF’s legal and organisational layers by ensuring transparent security obligations among partners.
• **MIM Sharing Data** (*Formerly known as ‘MIM 3 Contracts’*): Document analysis indicated that this MIM supports transparent usage control, provenance tracking, and service-level clarity. It provides contractual mechanisms to formalise interoperability agreements, anchoring governance practices in legal interoperability frameworks.

**Application-Level MIMs**

• **MIM Personal Data** (*Formerly known as ‘MIM 4 Trust*’): Workshop discussions revealed that experiments integrating frameworks such as MyData and SOLID, enable citizens to control access to their personal information while maintaining interoperability with public services. Survey results highlighted this MIM as crucial for transparency and user trust in citizen-facing applications.
• **MIM Geospatial Data** (*Formerly known as ‘MIM 7 Places’*): Project documentation demonstrated that this MIM supports the integration of geospatial datasets – including building footprints, mobility data, and environmental sensor readings – within digital-twin prototypes. This improved coordination between city planners, mobility operators, and utilities, showing how spatial data alignment strengthens cross-sector collaboration.
• **MIM Interoperable AI**: This MIM was not in the scope of the DS4SSCC analysis.
• **MIM Local Digital Twi**n: This MIM was not in the scope of the DS4SSCC analysis.

Across both foundational and application MIMs, DS4SSCC framework related results indicate that MIM maturity levels vary. Foundational MIMs (Data Access, Representing, Interlinking, Securing, Sharing) generate immediate operational benefits, such as faster data exchange, reduced integration costs, and improved compliance. Application-level MIMs Personal Data, Geospatial, Interoperable AI, Local Digital Twin) require greater coordination and yield strategic, cross-domain benefits, including improved transparency, innovation, and data reuse.

Persistent challenges included: (1) Uneven conformance documentation, which makes it difficult to verify consistent MIM implementation; (2) Varying technical and institutional capacities among local administrations; and (3) A lack of shared test suites and certification mechanisms, limiting the ability to assess alignment prior to deployment. Here, workshop participants suggested developing shared conformance frameworks, open testing environments, and procurement guidance to ensure consistent MIM application and measurable interoperability outcomes.

To conclude, the DS4SSCC experience demonstrates that long-term interoperability depends on balancing stable foundational mechanisms with flexible, adaptive application MIMs. Foundational MIMs secure technical and legal interoperability, while application-level MIMs enable innovation across domains such as AI, mobility, and digital twins. Together, these results show that MIMs Plus functions as both a reference framework and an operational toolkit – providing public administrations with a structured yet adaptable pathway to design, govern, and scale interoperable data spaces across Europe.

## 4 Discussion & Conclusions

The DS4SSCC experience demonstrates that interoperability in public-sector digital transformation is not a static technical achievement but an adaptive governance capability. This interpretation aligns with previous research highlighting that interoperability relies on institutional coordination, organisational learning, and capacity development as much as on technical design [[11], [12], [13]].

Through the five detected operational pathways—modular data architecture, shared and transparent governance, lightweight semantic alignment, procurement as a lever, and capacity building – DS4SSCC translated the European Interoperability Framework (EIF) into practical implementation structures. These pathways reflect the EIF’s layered logic across legal, organisational, semantic, and technical dimensions [15], showing how interoperability emerges through the alignment of architecture, governance, and institutional processes. The MIMs Plus framework provides the operational bridge that connects these layers, turning conceptual principles into actionable mechanisms guiding data management, governance coordination, and stakeholder collaboration.

The analysis found that foundational MIMs (Accessing, Representing, Interlinking, Securing, Sharing) deliver immediate improvements in data exchange, compliance, and trust, while application-level MIMs (Personal Data, Geospatial, Interoperable AI, Local Digital Twin) extend these capabilities into cross- domain innovation. This layered evolution supports prior findings that interoperability matures cumulatively through stable technical baselines and adaptive learning [14,18]. However, challenges persist: uneven local capacities, lack of shared conformance and certification frameworks, and rigid procurement procedures still limit consistent implementation and scaling across municipalities. These findings underscore that sustainable interoperability depends on institutional alignment and long-term capacity-building ecosystems. Shared testbeds, harmonised procurement guidance, and continuous professional training can strengthen coherence between frameworks and operational practices, ensuring the persistence of interoperability beyond project lifecycles.

In conclusion, the DS4SSCC project (preparatory action) carried out during the years 2022-2023 provides a working demonstration of Europe’s layered interoperability vision. By integrating EIF principles with the operational mechanisms of MIMs Plus, it reframes interoperability as a living, collective capability – grounded in governance, strengthened through collaboration, and continually adapted through institutional learning. Through the detected five operational pathways, interoperability moves from being a compliance requirement to becoming a strategic capacity essential for building sustainable, inclusive, and interoperable digital public services across Europe.

Future research should investigate how interoperability capabilities evolve over time and how contextual factors — such as governance culture, administrative traditions, national interoperability regimes, and local community engagement norms and capacities — shape the uptake of MIMs. In particular, studying the use and interaction of individual MIMs within the broader MIMs Plus framework in DS4SSCC-DEP (2023–2026) [62]— which aims to validate the DS4SSCC blueprint through 11 territorial, place-based pilot implementations — would offer valuable insight into how these mechanisms perform in practice and adapt to local dynamics. Such comparative and longitudinal studies could clarify the pathways through which interoperability matures institutionally, revealing how local communities co-shape technical, semantic, governance and contractual alignment, and help to refine both policy guidance and technical frameworks for future European data spaces.

## Ethics Statement

This study adheres to ethical guidelines, including GDPR compliance and informed consent where applicable, ensuring the protection of personal data.

## Declaration of Generative AI and AI-Assisted Technologies in the Writing Process

During the preparation of this work the author(s) used OpenAI. (2024). ChatGPT4.0 [Large language model]. in order to improve the readability and language of the manuscript. After using this tool, the authors reviewed and edited the content as needed and take full responsibility for the final version of the published article.

## CRediT authorship contribution statement

**Sophie Meszaros:** Conceptualization, Methodology, Investigation, Writing – review & editing. **Anniki Puura:** Methodology, Investigation, Writing – review & editing. **Thimo Thoeye:** Conceptualization, Writing – review & editing.

## Data Availability

While the reports are openly available (and cited in the text) the raw survey data is not available for publishing due to privacy concerns. Results of the 2023, March Stakeholder Forum are also restricted to Stakeholder Forum Members - one can become a stakeholder forum member by applying on ds4sscc.eu/stakeholderforum.
